# Hepatitis B surface antigen: carcinogenesis mechanisms and clinical implications in hepatocellular carcinoma

**DOI:** 10.1186/s40164-025-00642-7

**Published:** 2025-03-26

**Authors:** Bingyan Hao, Yachong Liu, Bohan Wang, Haofeng Wu, Yan Chen, Lei Zhang

**Affiliations:** 1 Hepatic Surgery Center, Institute of Hubei Key Laboratory of Hepato-Pancreato-Biliary Diseases, Tongji Hospital, Tongji Medical College, Huazhong University of Science and Technology, Wuhan, 430030 China; 2 Department of Paediatrics, Wuhan Union Hospital, Tongji Medical College, Huazhong University of Science and Technology, Wuhan, 430022 China; 3 Department of Hepatobiliary Surgery, Shanxi Bethune Hospital, Shanxi Academy of Medical Sciences, Shanxi Tongji Hospital, Tongji Medical College, Shanxi Medical University, Huazhong University of Science and Technology, Taiyuan, 030032 China; 4 Department of Surgery, Tongji Hospital, Tongji Medical College, Huazhong University of Science and Technology, Wuhan, 430030 China

**Keywords:** Hepatocellular carcinoma, Hepatitis B surface antigen, Oncoprotein, Tumorigenesis, Biomarker

## Abstract

Liver cancer is the third leading cause of death globally, with hepatitis B virus (HBV) infection being identified as the primary risk factor for its development. The occurrence of HBV-related hepatocellular carcinoma (HCC) is attributed to various mechanisms, such as chronic inflammation and liver cell regeneration induced by the cytotoxic immune response triggered by the virus, abnormal activation of oncogenes arising from HBV DNA insertion mutations, and epigenetic alterations mediated by viral oncoproteins. The envelope protein of the HBV virus, known as hepatitis B surface antigen (HBsAg), is a key indicator of increased risk for developing HCC in HBsAg-positive individuals. The HBsAg seroclearance status is found to be associated with recurrence in HCC patients undergoing hepatectomy. Additional evidence indicates that HBsAg is essential to the entire process of tumor development, from initiation to advancement, and acts as an oncoprotein involved in accelerating tumor progression. This review comprehensively analyzes the extensive effects and internal mechanisms of HBsAg during the various stages of the initiation and progression of HCC. Furthermore, it highlights the importance and potential applications of HBsAg in the realms of HCC early diagnosis and personalized therapeutic interventions. An in-depth understanding of the molecular mechanism of HBsAg in the occurrence and development of HCC is provided, which is expected to develop more precise and efficient strategies for the prevention and management of HCC in the future.

## Introduction

Liver cancer is the sixth most commonly diagnosed malignant tumor worldwide, with the third highest mortality rate [1]. Hepatocellular carcinoma (HCC), comprising approximately 75–85% of cases, is the predominant pathological type of liver cancer [1, 2]. Various risk factors contribute to the development of HCC, including long-term heavy drinking, HBV infection, hepatitis C virus infection, non-alcoholic fatty liver disease, obesity, diabetes, and metabolic syndrome [3]. HBV infection serves as the predominant etiological factor for HCC, contributing to over 40% of worldwide instances and in regions such as South-East Asia and Africa. This percentage rises to 60% or more [1, 3–8].

Current expert consensus guidelines indicate that HBsAg seroclearance is the ideal treatment endpoint for patients with chronic HBV infection [9–12]. HBsAg seroclearance is defined as at least two consecutive negative serum tests for HBsAg with an interval of more than six months, and this is considered a “functional cure” for chronic hepatitis B (CHB) [13, 14]. To date, multiple cohort studies verified and evaluated the feasibility of utilizing HBsAg clearance as the primary treatment endpoint [15–20]. The data indicated that the incidence of HCC in patients who achieved HBsAg seroclearance was significantly lower than in those with persistently positive HBsAg, and was comparable to the incidence observed in the HBsAg-negative cohort [18]. Furthermore, the method by which HBsAg is cleared, such as spontaneous clearance and therapy-induced clearance, did not affect the long-term clinical outcomes of patients [17]. It is evident that the HBsAg seroclearance is crucial for reducing the incidence of HCC.

In current clinical practice, radical surgical resection remains the preferred therapeutic approach for patients with HCC who exhibit preserved liver function and resectable tumors [21]. However, the incidence of postoperative recurrence persists at a substantial rate of 60–70% [22]. Huang et al. [23] found a notable correlation between elevated levels of preoperative HBsAg and an augmented risk of HCC recurrence after surgical resection. Similarly, Yoo et al. [24] reported a significant association between HBsAg seroclearance and a reduced risk of late recurrence of HCC postoperative. Regrettably, a thorough investigation into the potential association between specific levels of quantitative HBsAg and the risk of HCC recurrence was unattainable due to the absence of quantitative HBsAg testing in the routine assessment project of this cohort study. This highlights the necessity for further research to address this problem.

Numerous studies consistently found that patients with persistent HBsAg positivity face a significantly heightened risk, exceeding 5–10 times, of developing HBV-related HCC in comparison to those with HBsAg seroclearance [16, 18, 25]. These findings have motivated our study into the intricate molecular mechanisms underlying HBsAg-induced carcinogenesis and its potential implications for the onset, advancement, and postoperative recurrence of HCC. This review provides an overview of the oncogenic pathways activated by HBsAg, the signaling molecules regulated by HBsAg, and the role of HBsAg in tumor development. Furthermore, it highlights the significance of HBsAg as a crucial biomarker for distinguishing different clinical stages in chronic hepatitis and predicting the high risk of HCC development. Understanding this function of HBsAg is essential for determining treatment timing, thus preventing the occurrence and postoperative recurrence of HCC.

## The structure of HBV and expression of HBsAg

HBV, a type of DNA virus, belongs to the Hepadnaviridae family [26, 27]. Under an electron microscope, the complete HBV particles exhibit spherical morphology and consist of two distinct components: the outer shell and the inner core. The shell is composed of HBsAg, glycoproteins, and lipids, while the core particle primarily contains core protein (HBcAg) and the HBV genome [28].

**Fig. 1:**
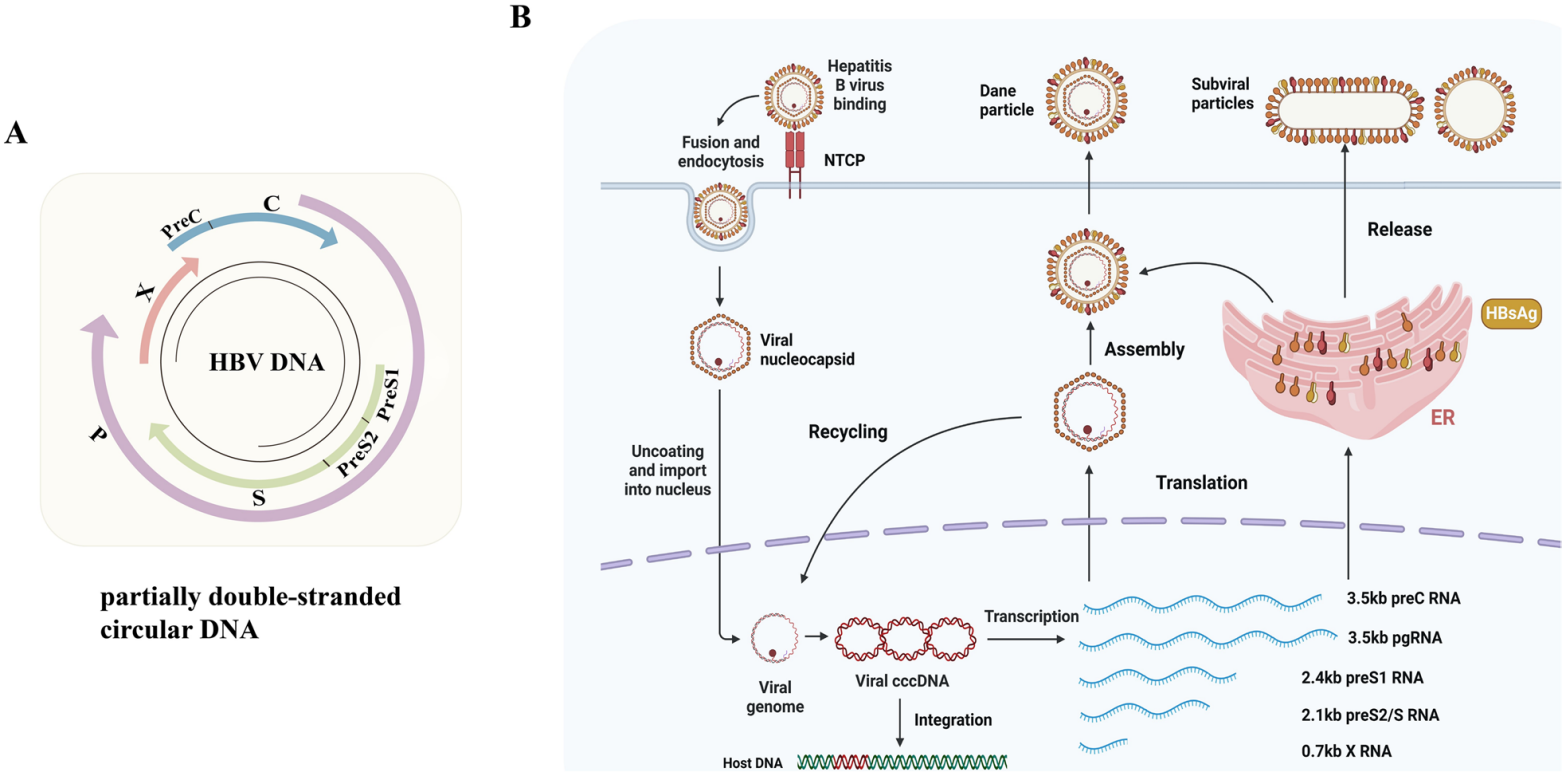
Schematic representation of the HBV viral genome (**A**), and the integration of the virus with infected hepatocytes (**B**). The virus binds to the NTCP receptor on hepatocytes and enters via fusion and endocytosis. Inside, the viral nucleocapsid is released into the cytoplasm and transported to the nucleus, forming cccDNA. This cccDNA serves as the template to produce five types of RNA, which move to the cytoplasm for protein translation. The pgRNA is packaged into core particles and undergoes reverse transcription to generate RC DNA. Mature core particles can either combine with envelope proteins to form Dane particles for release or return to the nucleus to produce more cccDNA. Furthermore, a significant quantity of envelope proteins, which are not bound to the core particles, is released from the cell as subviral particles. These particles participate in the processes of ongoing viral infection and immune modulation. cccDNA, covalently closed circular DNA; NTCP, sodium taurocholate co-transporting polypeptide; pgRNA, pre-genomic RNA; RC DNA, relaxed circular DNA

The HBV genome is composed of a partially double-stranded circular DNA with lengths from 3182 to 3248 base pairs, with variations dependent on genotype. This genome contains four functionally distinct open reading frames located on the same strand: preC/C, X, P, and preS/S, which collectively encode seven polypeptides [29] (Fig. 1). The preC/C gene features two in-frame start codons, encoding viral precore protein and the core protein. The open reading frame of the precore gene codes a hydrophobic leader peptide that guides the precore protein to the endoplasmic reticulum to be cut and form the secreted HBeAg [30]. The *X* gene encodes a 154-amino acid regulatory protein known as HBx, which lacks DNA-binding activity and contributes to carcinogenesis through various mechanisms such as transcriptional transactivation. HBx is the most extensively studied oncoprotein of HBV. The largest gene, the *P* region, completely overlaps with the envelope gene and partially overlaps with the *preC/C* gene and *X* gene. The protein encoded by the P region shows essential activities such as DNA polymerase, reverse transcriptase, and ribonuclease H, which are crucial for viral replication. The *pre-S/S* gene encompasses three in-frame start codons, leading to the division of pre-S/S into three distinct domains: Pre-S1, Pre-S2, and S structures. These domains collectively encode three envelope proteins – large envelope protein (LHB, 389-400aa), middle envelope protein (MHB, 281aa), and small envelope protein (SHB, 226aa)—collectively known as HBsAg [31]. These envelope proteins are primarily differentiated based on their structural domains and glycosylation status. The carboxyl-terminal S domain, comprising 226 amino acids, includes the N-terminal region (aa1–98), the main hydrophilic region (aa99–169), and the C-terminal region (aa170–226). This domain is responsible for constituting SHB, the main component of the virus envelope; the PreS2 domain, consisting of 55 amino acids, collaborates with the S domain to form the MHB, which is involved in the recognition of immune epitopes by T and B lymphocytes; the amino acid length of the PreS1 domain is variable, ranging from 108 to 119 amino acids depending on the subtype. The Pre-S1, Pre-S2, and S domains collectively form the LHB. The PreS1 domain can interact with the sodium taurocholate co-transporting polypeptide (NTCP) receptor found on hepatocytes, which is crucial for virus infection to enter host cells [32–34].

Infectious HBV particles attach to NTCP receptor located on the surface of hepatocytes through the preS1 domain of HBsAg. This interaction facilitates the entry of the viral core particles into the cytoplasm via endocytosis. Upon entry into the nucleus, the nucleocapsid disintegrates, releasing a relaxed circular form of double-stranded DNA (rcDNA). This rcDNA genome is repaired into a more stable covalently closed circular DNA (cccDNA), which binds to the host histones to establish a minichromosome [35]. This minichromosome serves as a template for the persistent transcription of viral RNA by HBV. Currently, there are no effective methods for its eradication. The cccDNA generates five distinct transcripts: the 3.5 kb preC mRNA and pgRNA, the 2.4 kb preS1 mRNA, the 2.1 kb preS2/S mRNA, and the 0.7 kb X mRNA. These transcripts were subsequently translated into seven viral proteins [36]. The hepatitis B virus (HBV) polymerase engages with the pre-genomic RNA (pgRNA) concurrently with the interaction of the core protein with the pgRNA, thereby initiating the assembly of a new nucleocapsid. Within the nucleocapsid, the pgRNA is encapsulated and undergoes reverse transcription to form the negative strand of HBV DNA, thereby commencing replication and ultimately generating a new nucleocapsid. This nucleocapsid may either be transported back to the nucleus to replenish the cccDNA pool or associate with envelope proteins that accumulate in the endoplasmic reticulum (ER) to form fully assembled viral particles for secretion. It is noteworthy that the excessive production of envelope proteins within the endoplasmic reticulum leads to the formation of spheroid or filamentous subviral particles (SVPs) lacking nucleocapsids. This process contributes to the associated pathological mechanisms and facilitates the development of HCC.

## Oncogenic roles of HBsAg

HBsAg, a surface protein of HBV, plays a critical role in the specific recognition of virus-infected cells and is intricately associated with the pathogenesis of HBV-related HCC. Research shows that individuals who tested positive for HBsAg had a significantly higher risk, ranging from 20 to 30 times, of developing HCC compared to those who were negative [37, 38]. Here, we offer an overview of the oncogenic properties and underlying molecular mechanisms of HBsAg in HBV-related HCC, encompassing cell proliferation, ER stress, metabolic reprogramming, immunity suppression, and tumor stemness regulation.

### HBsAg and proliferation

**Fig. 2:**
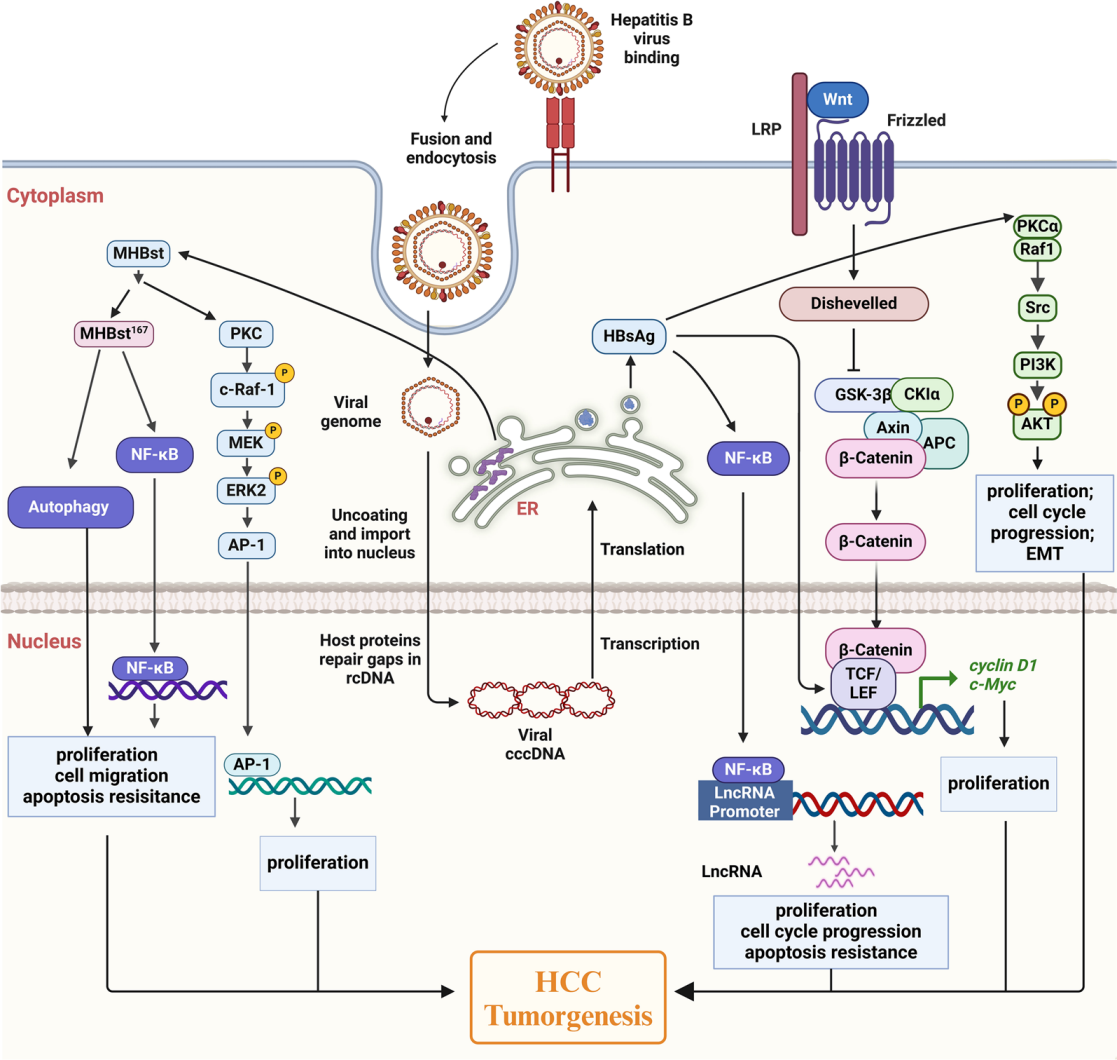
HBsAg induces the HCC tumorigenesis by promoting proliferation. After HBV infects hepatocytes, the envelope protein produced induces more active cell proliferation through multiple intracellular signal transduction pathways to promote HCC tumorigenesis, including: **a** activate the Src/PI3K/Akt pathway through proximal stimulation of PKCα/Raf1 signaling; **b** upregulate cyclin D1 and c-Myc through increasing the expression of LEF-1 in the WNT signaling pathway; **c** upregulate long non-coding RNA through the activation of the NF-κB signaling pathway; **d** trigger the PKC-dependent activation of the c-Raf-1/MEK/Erk 2 signaling pathway to regulate the AP-1; **e** induce autophagy and NF-κB activation. PI3K, phosphoinositide-3-kinase; PKC, protein kinase C; LEF-1, lymphoid enhancer-binding factor 1; MEK, mitogen-activated extracellular signal-regulated kinase; Erk 2, extracellular signal-regulated kinase 2; AP-1, activator protein 1

To date, research has found that HBsAg plays a role in promoting cell proliferation in HBV-associated HCC through various mechanisms (Fig. 2). The WNT signaling pathway is a crucial pathway linked to HCC, with lymphoid enhancer-binding factor 1 serving as a key component [39]. HBsAg was found to remarkably increase the expression of lymphoid enhancer-binding factor 1 in both tumor and adjacent tissues, resulting in the up-regulation of cyclin D1 and c-Myc*.* This contributed to more active cell proliferation and enhanced the malignant transformation of hepatocytes [40]. However, Tian et al. [41] studied a study on the HepG2-S-G2 cells overexpressing HBsAg, and found that the expression level of the c-myc and cyclin D1gene did not increase. Further research is still needed to determine whether there is a correlation between HBsAg and the upregulation of cyclin D1 and c-myc gene expression. Moreover, HBsAg was shown to induce HCC through the activation of the NF-κB signaling pathway to up-regulate long non-coding RNA LINC00665, which had the function of promoting cell proliferation, colony formation, cell migration, and inhibiting apoptosis [42]. Apoptosis is posited to have a dualistic role in the pathogenesis and progression of liver cancer. It can inhibit tumor development by facilitating the removal of malignant hepatocytes. Conversely, apoptosis may induce compensatory cellular proliferation by promoting the apoptosis of infected cells, thereby potentially advancing tumor progression. The influence of apoptosis in HCC is likely to be contingent upon several factors, including the presence of HBV infection, the immune response of the host, and the characteristics of the tumor microenvironment. Analysis of clinical data revealed a positive correlation between elevated levels of lncRNA DBH-AS1 and both tumor size and HBsAg. Subsequent investigations into the role of DBH-AS1 in HCC indicated that its overexpression significantly promoted cell proliferation and tumorigenesis [43, 44].

Among the three envelope proteins encoded by the HBV genome, studies found that LHBs are capable of inducing cell proliferation through activation of transcription factors, including activator protein 1 (AP-1) and nuclear factor-kappa B (NF-κB), which are known to be implicated in inflammation-induced tumorigenesis [45–47]. Liu et al. [48] further explored the role of LHB in hepatocellular tumorigenesis and revealed that LHBs activated the Src/PI3K/Akt pathway through proximal stimulation of PKCα/Raf1 signaling, thereby inducing cell proliferation and tumor formation. Additionally, LHBs could accelerate the progression of G1-S cell cycle and produce apoptosis resistance through the Src activation in HCC cells.

The presence of 3-terminally deleted PreS/S sequences in viral integrants of HBV-associated HCC was observed, resulting in the production of functionally active MHBst. This C-terminal truncation form of the medium surface antigens of HBV acts as a transcriptional activator protein, exhibiting tumor promoter-like properties that can transactivate oncogenes associated with tumors, ultimately leading to tumorigenesis [49–51]. In MHBst transgenic mice and HCC cells, protein kinase C (PKC)-dependent activation of the c-Raf-1/MEK/Erk 2 signaling pathway was triggered by the retention of MHBst in the ER. This led to the regulation of AP-1 and the enhancement of liver cell proliferation [45]. Furthermore, MHBst^167^, a special form of MHBst in which codon 167 is mutated to the stop codon, in immortalized cells was studied. The results showed that it could induce autophagy and NF-κB activation to promote cell proliferation, the cell cycle transition from the S phase to the G2/M stage, and the epithelial-mesenchymal transition process to promote the progression of HCC [52].

### HBsAg and ER stress

**Fig. 3:**
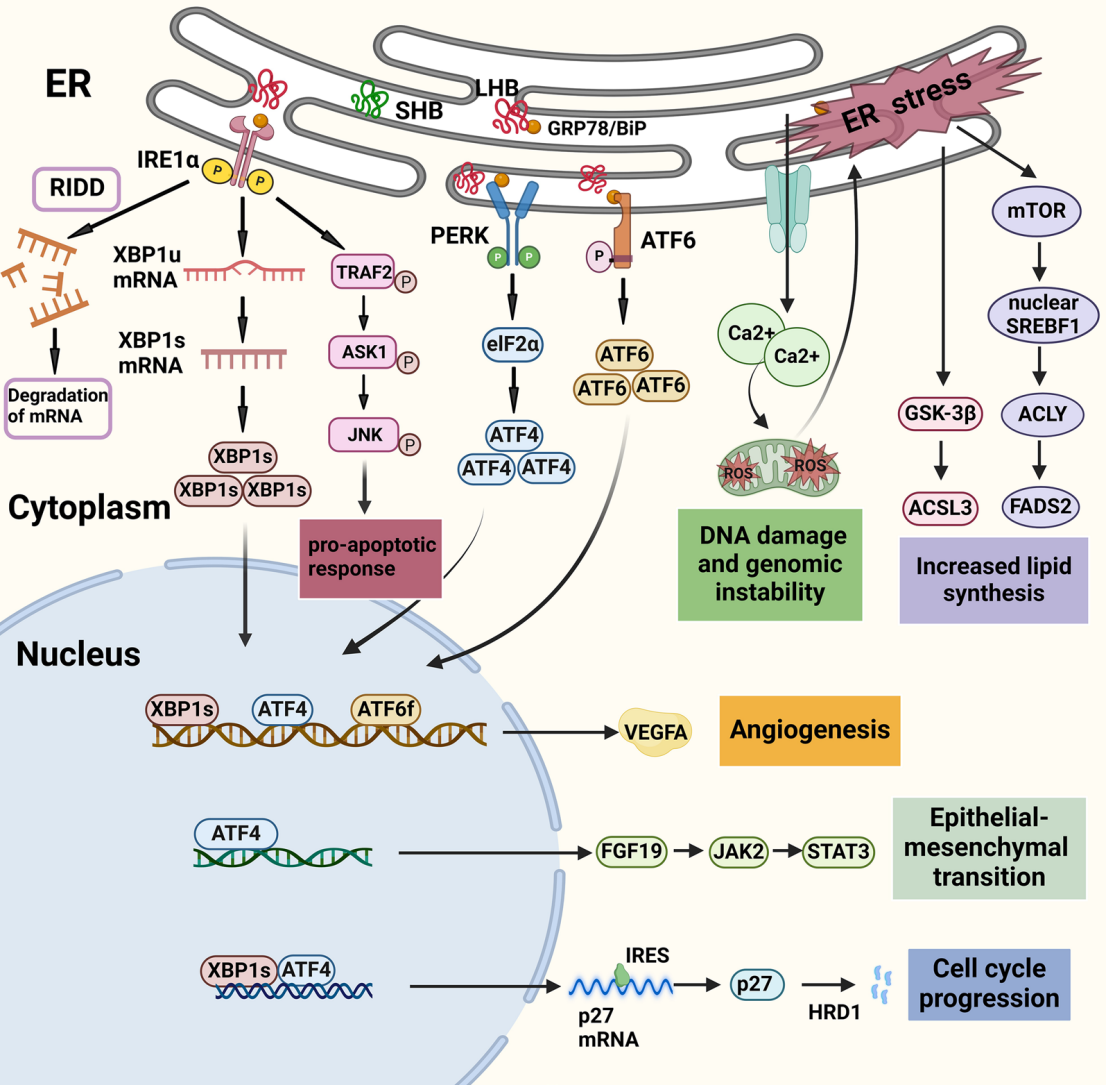
Various oncogenic effects induced by the ER stress in the development and progression of HBV-related HCC. Misfolding or imbalanced production of three HBV envelope proteins leads to their accumulation in the ER, inducing ER stress, which then causes cell cycle progression, epithelial-mesenchymal transition, angiogenesis, DNA damage, genomic instability, and increased lipid synthesis through different signal pathways, ultimately causing the development of HBV-HCC. ER, endoplasmic reticulum

The ER serves as the primary site for intracellular processes of protein folding, maturation, and transportation [53, 54]. Within the ER cavity, a diverse array of polypeptide chains and nascent proteins in various states of conformation are present [54]. In the presence of detrimental factors such as viral infection, aberrant protein expression resulting from genetic mutations, and cellular nutrient deficiencies, proteins may undergo misfolding or unfolding [55]. The ER stress is a cellular response mechanism where cells activate signaling pathways like unfolded protein response, ER overload response, and caspase-12-mediated apoptosis pathway when faced with pathological conditions involving misfolded and unfolded proteins [56]. Sustained ER stress can lead to the activation of oncogenic pathways, which in turn promote malignant transformation, proliferation, metabolic reprogramming, and alterations in the tumor microenvironment [57–59] (shown in Fig. 3). In a transgenic mouse model expressing HBV surface proteins, an imbalance in the production of LHB relative to SHB leads to the accumulation of these surface polypeptides and SVP particles lacking the HBV genome within the ER, leading to ER stress. This cascade of events causes chronic liver cell damage, abnormal proliferation, transcriptional dysregulation, carcinogenic aneuploidy lesions, and ultimately contributes to an increased incidence of HCC [60].

Previous studies showed that the accumulation of LHB in the ER led to sustained ER stress, activating the unfolded protein response (UPR) and ER overload response. The unfolded protein response encompasses three primary signaling pathways: the inositol-requiring enzyme 1α (IRE1α) pathway, the protein kinase R-like endoplasmic reticulum kinase (PERK) pathway, and the activating transcription factor 6 (ATF6) pathway [61]. Initially, IRE1α undergoes phosphorylation, facilitating the splicing of X-box binding protein 1 (XBP1) mRNA to generate the active transcription factor XBP1s, which subsequently enhances the transcription of downstream oncogenes. Concurrently, IRE1α can directly degrade specific mRNA substrates via the regulated IRE1-dependent decay (RIDD) process, thereby promoting the survival of tumor cells. During endoplasmic reticulum stress, IRE1α engages with tumor necrosis factor receptor-associated factor 2 (TRAF2) via its kinase activity, thereby initiating the activation of apoptotic signal-regulating kinase 1 (ASK1), which subsequently leads to the phosphorylation of c-Jun N-terminal kinase (JNK). The activated JNK facilitates apoptosis by further activating c-Jun and promotes the expression of inflammatory genes by AP-1 through the phosphorylation of transcription factors [56, 61–63]. The activation of the PERK pathway occurs in a manner analogous to that of IRE1α. Upon phosphorylation, PERK activates the downstream target eIF2α, which subsequently enhances the translation of transcription factor ATF4, thereby initiating transcription of a series of downstream genes [64]. Following its dissociation from glucose-regulated protein 78 (GRP78/BiP), ATF6 translocates from the endoplasmic reticulum to the Golgi apparatus, where it undergoes proteolytic cleavage to generate transcription factor ATF6f and then enters the nucleus to facilitate gene transcription [65–67]. The ER overload response triggered Ca^2+^ efflux from the ER lumen and the release of reactive oxygen species from mitochondria, causing oxidative DNA damage and genomic instability [66]. These effects collectively contributed to the development of HCC. Research has found that LHB could initiate UPR signaling directly in the context of cell proliferation, leading to sustained ER stress. This stress condition resulted in selective translation of P27 oncogene mRNA, followed by ubiquitination and degradation of the translation product, P27 protein, by the E3 ubiquitin ligase HRD1. The decrease in P27 protein levels facilitated the progression of cell cycles by diminishing G1/S phase arrest, contributing to the development of HCC [68].

The upregulation of SHBs has been shown to facilitate HCC progression through the induction of ER stress. Wu et al. [69] discovered that the expression of SHB in HCC cells triggered the activation of ATF 4, leading to elevated expression and secretion of fibroblast growth factor 19 via ER stress. The release of fibroblast growth factor 19 subsequently activated the JAK 2/STAT 3 signaling pathway, promoting the epithelial-mesenchymal transition in HCC cells and significantly enhancing their migratory, invasive, and metastatic capabilities [70]. Moreover, SHB activated the UPR through a triad of ER membrane-resident sensors consisting of IRE1α, PERK and ATF6. Interestingly, their respective transcription factors XBP1s, ATF4, and ATF6f possessed consensus sites on the vascular endothelial growth factor A (VEGFA) promoter region to drive VEGFA transcription through binding to the promoter [71]. Consequently, this process resulted in increased expression and secretion of VEGFA, thereby enhancing the angiogenic capacity of HCC cells and microvessel density within tumors [72]. This finding indicated that the SHB played a significant role in promoting angiogenesis in HBV-related HCC, implying that it could serve as a promising target for anti-angiogenic therapy in the treatment of HCC.

ER stress disrupts bodily homeostasis via various signal transduction pathways and is intricately linked to the progression of severe liver disease and HCC. Therefore, if it is impossible to prevent the abnormal accumulation or misfolding of viral envelope proteins from causing ER stress, some feasible ways are considered to obstruct the activation of corresponding signaling pathways after ER stress, such as preventing the binding of the three sensors on the ER membrane and their downstream effector molecules or using structural analogs of GRP78 to hinder the phosphorylation of the three transmembrane proteins.

### HBsAg and metabolic reprogramming

Tumor metabolism refers to the utilization of diverse metabolic pathways by neoplastic cells to fulfill increased bioenergetic and biosynthetic demands [73, 74]. The metabolic process of cells is regulated by a variety of mechanisms, including aberrant expression of particular metabolites, oxidative stress environment, and abnormal activation of oncogenes. Abnormal metabolic alterations create a conducive environment for the proliferation, survival, and metastasis of tumor cells, ultimately advancing the progression of HCC by facilitating tumor reprogramming [74].

Carbohydrate metabolism is an intricate biological process, and dysregulation of glucose metabolism is a significant factor in the initiation and progression of HCC. Research has discovered that LHBs interacted with pyruvate kinase isoform M2 and inhibited its function, prompting a shift in hepatocyte metabolism from typical oxidative phosphorylation to aerobic glycolysis, leading to heightened glucose consumption and lactate production. This metabolic alteration was believed to support viral protein synthesis and promoted virus-induced liver carcinogenesis [75]. Chen and colleagues demonstrated a novel finding that SHB of HBV upregulated the transcription and expression of adenylyl cyclase 1 via the binary E-box factor binding site, leading to the activation of cAMP/PKA/CREB signaling pathway. This subsequently enhanced the transcription and expression of gluconeogenesis genes, including glucose-6 phosphatase and phosphoenolpyruvate carboxykinase, ultimately promoting hepatic gluconeogenesis [76]. The study unveiled the novel pathogenic function and mechanism of SHB in hepatic gluconeogenesis, offering promising targets for the prevention and treatment of glucose metabolism disorders in individuals with HBV infection. Nevertheless, additional research is warranted to reveal the precise mechanism by which SHBs modulate the transcriptional activity of adenylyl cyclase 1 via nuclear receptors/E-box binding factors and binary E-box factor.

Furthermore, HBsAg has been found to affect glucose metabolism by activating the ER stress signaling pathways. This activation led to the ectopic of the molecular chaperone GRP78/BiP in the ER lumen, as well as the phosphorylation of three ER stress sensors/transmembrane proteins (IRE1α, PERK, and ATF6). The IRE1α/XBP1 branch was responsible for reducing glucose metabolism through modulating of hypoxia-inducible factor 1α and glucose transporters 1 and 2 [77–79]. The PERK/ATF 4 branch played a role in the regulation of amino acid biosynthesis, mitochondrial stress response, and glycolysis [80–82].

The liver plays an essential role in maintaining lipid homeostasis, with disruptions in lipid metabolism being considered to be the driving force of HCC. Gong et al. [83] found that the presence of SHBs in HCC cells, either transiently or stably expressed, resulted in a direct interaction with short-chain enoyl-CoA hydratase 1(ECHS1), leading to a significant decrease in ECHS1 protein levels. ECHS1 was an essential enzyme involved in the second step of the beta-oxidation pathway within mitochondrial fatty acid metabolism [84]. As a result, it was hypothesized by researchers that SHBs may contribute to the development of hepatic steatosis through the down-regulation of ECHS1 levels. However, the veracity of the hypothesis and the role of this protein interaction in HCC remained to be further investigated.

The preS1 domain of the large hepatitis B surface antigen (LHB) encompasses amino acid sequences that interact with the sodium taurocholate cotransporting polypeptide (NTCP) receptor [85, 86]. This receptor plays a crucial role in the uptake of the majority of sodium-dependent bile salts by hepatocytes. Yan et al. [87] identified potential overlapping regions between bile salts and the pre-S1 domain at the binding sites on the NTCP, suggesting mutual inhibition between the two. Specifically, the binding of the pre-S1 domain to human NTCP impedes the uptake of taurocholic acid by these receptors. Additionally, certain bile salts interacting with NTCP exert an inhibitory effect on the entry of HBV and hepatitis D virus (HDV) in a dose-dependent manner. Bile salts, synthesized by hepatocytes, constitute the primary components of bile and facilitate lipid digestion through emulsification within the small intestine. Beyond their digestive function, bile salts are integral to various physiological processes. Disruption of bile salt transport can lead to lipid metabolism disorders by affecting the farnesoid X receptor (FXR) signaling pathway and modulating the transcription of genes associated with lipid metabolism [88, 89].

Moreover, in the HBV transgenic mouse model, ER-stress induced activation of the mTOR signaling pathway, resulting in upregulation of ATP citrate lyase and fatty acid desaturase 2 via sterol regulatory element binding transcription factor 1 [90]. This led to elevated levels of triglycerides and cholesterol. Additionally, LHB could increase the expression of acyl-CoA synthetase through the ER stress-mediated glycogen synthase kinase signaling pathway, thereby promoting lipid synthesis [91].

### HBsAg and immune suppression

**Fig. 4:**
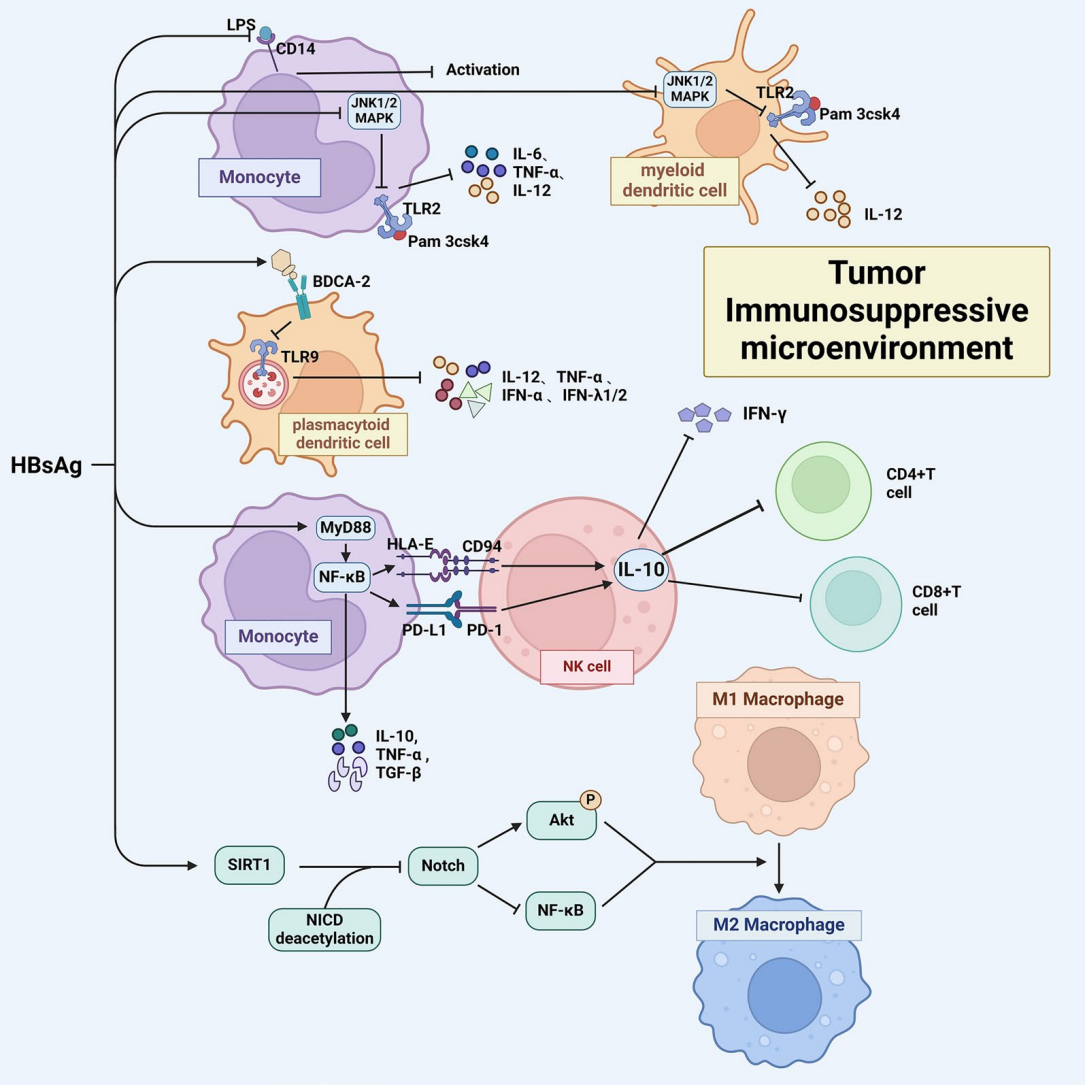
HBsAg suppresses the immune response by impairing the phenotype and function of immune cells. Normal immune function is crucial for killing tumor cells. HBsAg damages the immune system by inhibiting the functions of monocytes, dendritic cells, NK cells, and macrophages, which creates a suppressive tumor immune microenvironment, thereby conducive to the progression of HBV-related HCC. NK, natural killer

The immune system is the most powerful defense force of the human body, and the immune response of the body plays a key role in suppressing tumor development and progression [92]. Some studies found that HBsAg suppressed the immune response process in patients by impairing the phenotype and function of immune cells, thereby leading to immune tolerance and immune escape in HCC cells, ultimately facilitating tumor progression [93, 94] (Fig. 4). An in vitro experiment found that HBsAg could induce maturation disorders and dysfunction of myeloid dendritic cells in CHB patients [95]. Studies conducted at the onset of the twenty-first century revealed that recombinant yeast-derived HBsAg could interact with monocytes via lipopolysaccharide (LPS)-binding protein and LPS receptor CD14, resulting in the decrease of LPS-induced monocyte activation [96]. Also, HBsAg was found to diminish the production of LPS-induced tumor necrosis factor-α (TNF-α) by impeding the activation of ERK-1/2 and JNK-1/2 kinases [97]. In addition, HBsAg hindered the production of IL-12 by interfering with the JNK-MAPK pathway and inhibiting myeloid dendritic cells and monocytes/macrophages in response to Pam 3csk4 stimulation, resulting in a significant tolerogenic phenotype. At the same time, this interference adversely affected the T cell stimulatory capacity of dendritic cells, consequently impairing the type 1 helper T cell response [95, 98, 99].

HBsAg is associated with the dysfunction of plasmacytoid dendritic cells [100]. It could inhibit the secretion of cytokines, including IL-12, TNF-α, IFN-α, IFN-λ1, and IFN-λ2 through the down-regulation of toll-like receptor (TLR) expression and the suppression of TLR 9-mediated plasmacytoid dendritic cell maturation, thereby suppressing the immune system response and fostering a microenvironment conducive to cancer progression [101–103].

Natural killer (NK) cells, serving as the primary effector cells of the innate immune system, play a critical role in the defense against HBV virus infection. Research indicated that HBsAg has the potential to affect the phenotype and functionality of NK cells [93, 104–106]. In vitro studies show that HBsAg could impede the activation of NK cells, cytokine secretion, and release of cytotoxic substances via signal transducer and activator of transcription 1 (STAT 1) phosphorylation, and a negative relationship was observed between the activation and cytotoxicity of NK cells and the level of HBsAg [106–108]. Moreover, the presence of HBsAg in HCC cells infected with HBV stimulated inhibitory monocytes to release cytokines, including IL-10, TNF-α, and TGF-β via the MyD88/ NF-κB signaling pathway. Simultaneously, it also stimulated high expression of the inhibitory molecules programmed cell death ligand 1 (PD-L1) and major histocompatibility complex-E(HLA-E) in inhibitory monocytes, which triggered an immunosuppressive cascade through the interaction of PD-L1/PD-1 and HLA-E/CD94, leading to the generation of regulatory NK cells that release IL-10. These cells could suppress autologous NK cell activation and CD4^+^ and CD8^+^ T cell activation via an IL-10-dependent pathway, thus facilitating HBV immune evasion [109]. This phenomenon offers significant implications and creates conducive conditions for the persistence of HCC cells.

Monocyte-derived macrophages in the liver also modulate anti-tumor immune responses. Research indicated that HBsAg could inhibit the TLR2-induced phosphorylation of p38 and JNK/MAPK, leading to decreased production of pro-inflammatory cytokines such as IL-6, TNF-α, and IL-12 in human monocytes [98]. Moreover, in HCC cell lines expressing HBV DNA, HBsAg promoted the deacetylation of Notch 1 intracellular domain by upregulating the expression of sirtuin 1, contributing to the polarization of M2 macrophages and the induction of immune tolerance in tumor cells infected with HBV [110].

Sustained elevated levels of HBV antigens, specifically HBsAg and HBeAg, have been implicated in T cell depletion and compromised adaptive immune responses. Research indicates that HBV-related antigens, including HBcAg and HBsAg, can increase the expression of inhibitory molecules on CD4 + T cells, consequently leading to a diminished humoral immune response [111, 112]. Burton et al.[113] found that, despite the persistence of HBsAg-specific B cells in the blood and liver of patients infected with HBV, these B cells exhibited impaired antibody secretion capabilities. Furthermore, they revealed the CD21-CD27- atypical memory B cell (atMBC) phenotype and exhibited elevated expression of inhibitory receptors, including PD-1, BTLA, and CD22. This phenotype significantly compromised the normal adaptive immune response in these patients.

There is a strong correlation between the presence of HBsAg and the reactivation of HBV. In patients experiencing HBV reactivation, the S gene of HBsAg exhibits an unexpectedly high level of genetic diversity and complexity [114–118]. Mutations in the S gene predominantly occur in major hydrophilic regions, encompassing B cell and T cell epitopes, which may impede the recognition of S-HBsAg by antibodies [119, 120]. Moreover, an increased presence of additional N-linked glycosylation sites within the principal hydrophilic region of S-HBsAg has been observed in patients experiencing HBV reactivation, a phenomenon not observed in chronically infected individuals [119, 121, 122]. These N-linked glycosylation sites have the potential to obscure B cell epitopes, thereby concealing them from anti-HBs antibodies. This masking effect promotes the recognition and quantification of HBsAg, which is crucial for the virus's evasion of the humoral immune response [119, 122, 123].

### HBsAg and tumor stemness

Cancer stem cells represent a distinct subset of cells that possess unique attributes including self-renewal capacity, differentiation potential, high tumorigenicity, and high drug resistance. These cells are pivotal in the occurrence, metastasis, drug resistance, and recurrence of tumors [124, 125]. Anfuso et al. [126] observed progressive liver damage from chronic hepatitis to HCC in HBsAg-positive transgenic mice. In this process, the liver stem cell population was activated, causing corresponding alterations in its markers at various stages of liver injury. Specifically, the expression of CD 34 and Sca-1 was up-regulated in the early stages of injury, while the expression of Krt 19, Sox, Epcam, and CD 133 increased as the injury advanced. This series of changes indicated the importance of HBsAg as a carcinogen in the development of HCC. Furthermore, Liu et al. [127] studied the preS1 protein, preS2 protein, and HBx protein, revealing that the preS1 protein could induce the expression of cancer stem cell (CSC) markers CD133 and CD117 in normal liver cells. Additionally, the activation of CSC-related genes such as Klf4, Sox2, Nanog, c-Myc, and Oct4 was significantly increased, thereby facilitating the generation and self-renewal of cancer stem cells. This finding holds significant implications for identifying novel targets for the prevention and treatment of HCC related to HBV. At the epigenetic level, HBsAg can facilitate the progression of HCC and tumor stemness through substantially downregulating the expression of miR-203a, ultimately resulting in an unfavorable prognosis [128].

### Other mechanisms

In addition to the above mechanisms, HBsAg can also promote the development of HCC by inducing cytokinesis failure and upregulation of oncogenes. Li et al. [129] found that LHB could induce cytokinesis failure by causing DNA damage and polo-like kinase 1-mediated G2/M phase checkpoint failure in hepatocytes, leading to hyperploidy and facilitating HCC progression. This finding further revealed the underlying molecular mechanism of LHB-induced HCC development and identified potential targets for interfering with LHB-induced aneuploidy cycles, which may be helpful for patients with failed HBsAg serum clearance after antiviral treatment.

The preS2 region is present in both LHB and MHB, with previous research demonstrating its role in regulating gene expression through transactivation of AP-1 and other transcription factors [130]. A study found that the preS2 protein interacted with a 20-bp region known as the preS2-responsible region to transactivate the human telomerase reverse transcriptase (hTERT) promoter, leading to elevated up-regulated telomerase activity and facilitating HCC development [131]. Furthermore, overexpression of preS2 has been shown to enhance the telomerase activity, cell proliferation, and tumorigenicity in hepatocytes, thereby promoting the progression of HCC [131]. Moreover, the preS2 protein could increase the expression of forkhead box protein P3 (FOXP3) in malignant hepatocytes through transactivation of the FOXP3 core promoter [132].

## Oncogenic roles of HBsAg mutants

**Fig. 5:**
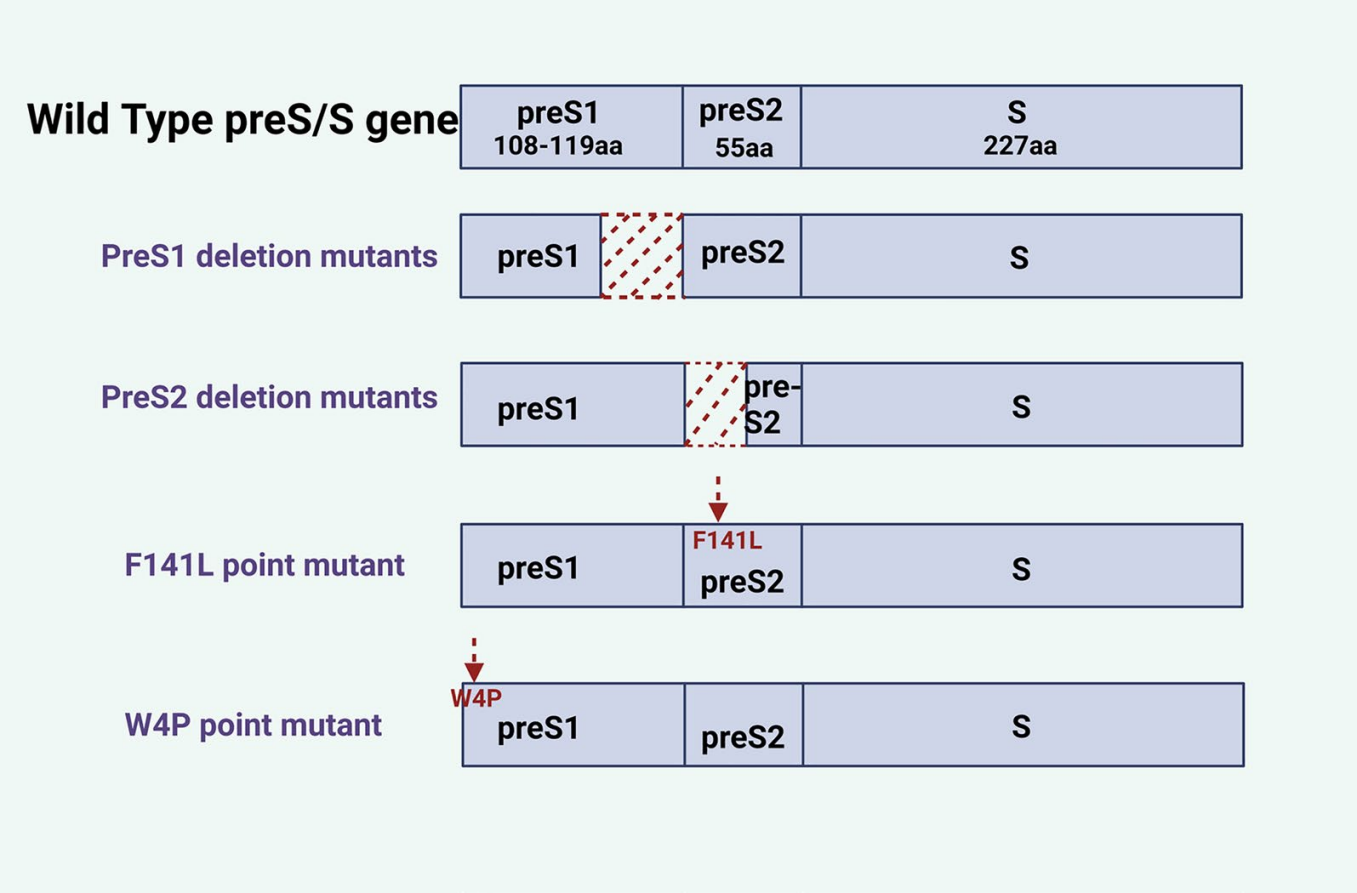
Schematic representation of multiple HBsAg mutants

**Fig. 6:**
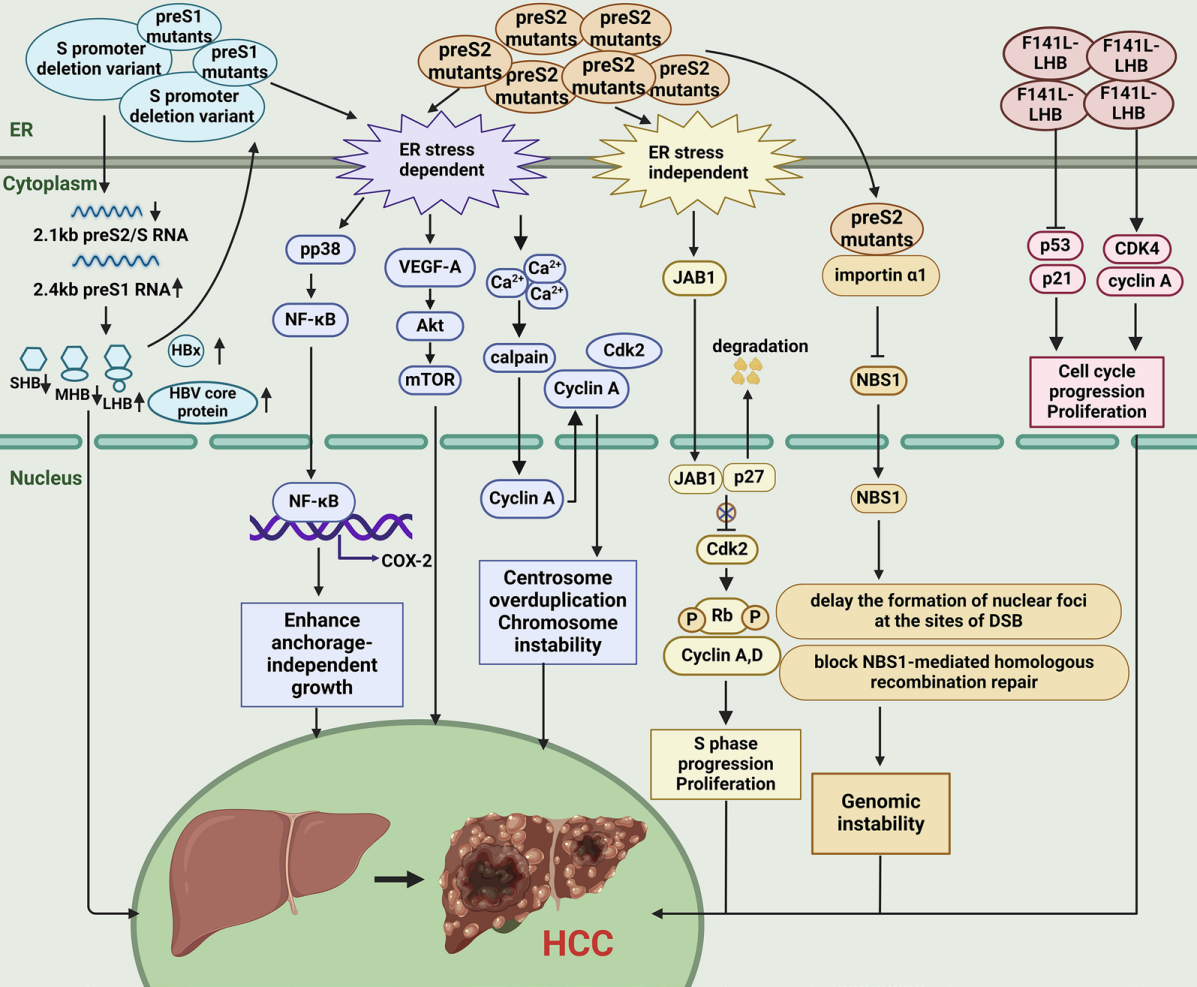
Oncogenic mechanisms of HBsAg mutants in hepatocarcinogenesis. Mutated envelope proteins accumulate in the endoplasmic reticulum and regulate cytoplasmic signaling pathways through ER stress-dependent and ER stress-independent signals, thereby causing proliferation, cell cycle progression, genome instability, and inducing the occurrence of HCC. In addition, when deletion mutations occur in the preS1 region, including deletion of the S promoter. It will affect the reduction of downstream 2.1 kb preS2/S RNA production and the relative increase in the 2.4 kb preS1 RNA production, resulting in an increase of HBx and HBeAg as well as an imbalance in the production of three surface proteins, thereby promoting the occurrence of HCC. ER, endoplasmic reticulum

Due to the spontaneous error rate of viral reverse transcription and the influence of host immunity or specific treatment, multiple HBV surface protein mutants will appear during patient infection, including preS1 deletion mutants, preS2 deletion mutants, F141L point mutant, W4P LHB mutant, etc. [34] (Fig. 5). Multivariate analysis revealed a significant association between the presence of preS mutants and an elevated risk of HCC [133]. Notably, preS2 deletion mutants exhibit potent oncogenic properties, being identified as a “precursor lesion of HCC” and a risk factor for postoperative recurrence of HCC [134–137]. In the following, we provide a summary of the presently understood mechanisms by which mutant forms contribute to cancer promotion (Fig. 6).

### Pre-S deletion mutants

Pre-S deletion mutants include two types of mutants: preS1 deletion mutants and preS2 deletion mutants. Research indicated that in-frame deletion of the 3´end of the preS1 region not only affected the coding of normal LHB but also greatly reduced SHB synthesis due to the deletion region containing the promoter of 2.1 kb-RNA encoding SHB. Meanwhile, these deletion mutants led to an increase in levels of HBx, core protein, and DNA replication within cells and hindered the normal secretion of viral proteins and particles, inducing the occurrence of HCC [138]. Additionally, HBV preS1 mutants could also promote hepatocarcinogenesis through the transactivation of the *TGF-α* gene [139].

The preS2 deletion mutant protein has been identified as a viral oncoprotein [131, 137, 140, 141]. It accumulates in the ER and presents as type II ground glass hepatocytes (GGH), which are recognized as the morphological hallmark of late-stage hepatocytes in chronic HBV infection and precancerous lesions [142, 143]. Studies reported that HCC patients harboring type II GGH exhibited increased rates of recurrence and mortality compared to those without this histological feature [137, 143].

Previous studies found that preS2-mutant LHBS could elicit oxidative DNA damage and genomic instability in hepatocytes via ER stress [137, 141]. The preS2 deletion mutant initiated the ER stress-dependent VEGF/Akt/mTOR signaling pathway and the NF-κB/COX-2 signaling pathway, which were activated in Huh-7 cells, thereby enhancing carcinogenesis of the HBx and HBsAg that simultaneously accumulated in GGH in transgenic mice and humans [144–146]. In addition to the ER stress-dependent signaling pathways, preS2 deletion mutants could also induce HCC through unique ER stress-independent responses. This mutant protein interacted directly with c-Jun activation domain-binding protein 1 (JAB 1), resulting in the degradation of the cyclin-dependent kinase inhibitor p27 and the hyperphosphorylation of the tumor suppressor retinoblastoma (Rb). This ultimately induced progression from the G1 to S phase of the cell cycle and augmented the transcriptional activity of activator protein-1 and cell proliferation [147]. Moreover, the interaction between the preS2-mutant protein and JAB 1 led to the nuclear translocation of JAB 1, resulting in the activation of the p27/retinoblastoma/Cdk 2/cyclin A and D pathway. This activation promoted cell cycle progression and centrosome overduplication, ultimately resulting in chromosomal instability in liver cells and significantly increasing the likelihood of gene mutations [34, 148].

Hsieh and colleagues performed additional research on the role of the preS2 deletion protein and discovered that the preS2-mutant LHBS retarded the DNA repair and the nuclear translocation of recombination factor Nijmegen breakage syndrome 1 by interacting with importin α1, thereby inhibiting the formation of nuclear foci at DNA double-strand break sites and Nijmegen breakage syndrome 1-mediated homologous recombination repair [149]. Furthermore, Wang et al. [150] found that the accumulation of preS2 mutant LHBS in the ER induced ER stress, triggering calcium release to activate the calcium-dependent calpain. Hydrolysis of this enzyme led to the redistribution of cytoplasmic cell cyclin A and excessive centrosome duplication, ultimately leading to the generation of aneuploidy and polyploidy. These events contributed to an elevation in global gene copy number variation and genome instability within hepatocytes, thereby facilitating the development of HCC.

### Other mutants

In addition to the extensively researched preS deletion mutant proteins, there are fewer frequently observed mutant forms within the preS/S region that also contribute significantly to the pathogenesis of HCC. Mun et al. [151] conducted a study involving 241 patients with chronic liver disease to determine a significant association between the presence of the F141L point mutant in the preS2 region and HCC. Subsequent in vitro experiments revealed that F141L-LHBs curbed the expression of p53 and p21 pathways, impairing cell cycle checkpoints in the G1/S phase and facilitating mutation accumulation, ultimately leading to the development of HCC. F141L-LHBs also upregulated cyclin-dependent kinase 4 and cyclin A, promoting cell cycle progression and enhancing cell proliferation. Furthermore, it is noteworthy that combinatorial mutations are frequently observed in patients with HCC. The combined mutations at the preS2 start codon (M120) and the F141L double mutation were shown to exhibit greater cell proliferation and colony formation capabilities than the single F141L mutation [151].

Research indicated that male gender is an independent risk factor for the development of HCC, with males having a three to five-fold greater risk compared to females [152–154]. Lee et al. [155] suggested that HBsAg mutants played a role in the sex-specific mechanism of tumorigenesis. The W4P LHB mutant occurring in the preS1 region potentially promoted the HCC progression in male CHB patients through the IL-6-dependent pathway. This study indicated that estrogen could inhibit W4P LHB-induced tumor growth in male mice by suppressing the production of IL-6. This discovery held significance in the evaluation of estrogen as a potential therapeutic option for male patients with HCC induced by W4P LHB.

At present, occult hepatitis B infection (OBI) is characterized by the presence of replicative HBV in the form of cccDNA within hepatic tissue and/or the detection of HBV DNA in the bloodstream of individuals who tested negative for HBsAg using current diagnostic assays [156]. OBI arises from diverse origins, encompassing individuals who have achieved clinical resolution of chronic hepatitis B, those who have been serologically cleared of HBsAg yet retain residual HBV within hepatic tissue, and individuals infected with mutant strains of HBV [157–159]. Numerous studies investigating HBV variants in the context of OBI have predominantly concentrated on the principal hydrophilic region of the S gene, mutations occurring within this region, such as G145R, modify the antigenic properties of HBsAg, rendering them undetectable by existing commercial HBsAg assays [119, 159].

Furthermore, alongside mutations in the S-ORF, numerous pre-S mutants, particularly deletion mutations in the pre-S1 and pre-S2 regions, have been shown to have a strong association with OBI, including the loss of half of the C-terminal of the pre-S1 region, the loss of the pre-S2/S promoter, and the loss or missense mutation of the pre-S2 start codon [122, 160–164]. These mutants have the potential to disrupt the synthesis of surface proteins, resulting in their retention within the ER of hepatocytes. The subsequent accumulation of these mutated surface proteins can precipitate ER stress, thereby inducing oxidative DNA damage, genomic instability, and an elevated risk of carcinogenesis [34, 165].

## Clinical significance of HBsAg

### The predictive potential of HBsAg for HCC progression in inactive HBV patients

Risk factors for HCC encompass various variables such as gender, age, HBV genotype, ALT level, hepatitis B e antigen status, and HBV DNA level [166]. High levels of HBV DNA can be a major driver of disease progression in CHB patients [167–169]. When the HBV DNA level is higher than, or equal to, 2000 IU/mL, there is a notable increase in the risk of HCC [170]. Conversely, individuals with HBV DNA levels below 2000 IU/mL are typically classified as inactive or low-risk HBV carriers [171–173]. However, there are currently no additional markers available to predict the risk of HCC in this patient population. A retrospective study reported a strong predictive value of HBsAg levels for inactive HBV infection [174]. Tseng et al. [166] found high HBsAg levels increased the risk of HCC in HBeAg-negative individuals with low viral loads. The study identified HBsAg levels greater than, or equal to, 1000 IU/mL as an independent risk factor for HCC development, suggesting a complementary relationship between HBsAg and HBV DNA levels in predicting HCC occurrence, particularly in HBV carriers with low viremia [175].

Furthermore, research suggested that the levels of HBsAg could serve as a marker for distinguishing between chronic infection and hepatitis stages [176–181]. In the clinical course of chronic hepatitis B, it is difficult to distinguish between the inactive carrier stage and the HBeAg-negative CHB stage, and the prognosis of the latter has a significantly higher risk of cirrhosis and HCC than the former [182, 183]. Therefore, identification of patients in the stage of inactive carrier and stage HBeAg-negative CHB is crucial for the rational treatment of chronic hepatitis B and for preventing the progression of hepatitis B to HCC. Studies further found that the ratio of LHB to MHB was more indicative of disease stage than the total HBsAg level. Specifically, a low LHB ratio is a more accurate predictor of the stage of inactive carrier than a low total HBsAg level [184]. Hence, quantification of HBsAg fractions could potentially serve as a novel approach to prevent the occurrence of HCC.

### PreS/S mutants as biomarkers for HCC risk prediction

HCC is a leading cause of cancer mortality, so continuous surveillance of HCC progression is also one of the important means of timely preventive treatment. Alpha-fetoprotein (AFP) is currently the most commonly used serum marker for clinical detection and follow-up of HCC. Nonetheless, approximately one-third of HCC patients will have normal AFP levels [185–187]. Consequently, there is a need to identify more dependable serum biomarkers to evaluate the risk of HCC progression. Numerous studies have demonstrated the oncogenic potential of preS mutants in HCC through both in vivo and in vitro experiments and played a significant role in carcinogenesis. Several retrospective studies explored the prevalence of preS gene deletion and revealed a notably higher prevalence in the blood of HCC patients compared to those with hepatitis B or cirrhosis [188–190]. Furthermore, Chen et al. [191] discovered that CHB patients with preS gene deletion exhibited a 5-year cumulative incidence of cirrhosis and HCC approximately five times higher than those without preS gene deletion. These data evinced the clinical correlation between preS gene deletion and HCC development.

The preS2 deletion mutant LHB was found to strongly correlated with HBV-related HCC and pathological analysis revealed an association between this mutant and the histological morphology of type II GGH [192, 193]. GGH II is recognized as a precursor lesion of HCC, and several studies indicated a heightened risk of tumor recurrence following radical liver resection in individuals with GGH [143]. Yen et al. [193] further studied the relationship between preS2-mutant LHBS levels in serum and HCC recurrence. The results showed that some patients had low serum preS2-mutant LHBS levels before liver resection, but they increased significantly during postoperative recurrence. In addition, in tissue staining in the peritumoral area, type II GGH intensity was found to be associated with HCC recurrence and subsequent mortality. This finding reaffirmed the crucial role of preS2-mutant LHBS in predicting the risk of HCC recurrence. Cox regression analysis indicated that the combination of serum preS2-mutant levels and AJCC tumor stage could reliably predict the risk of recurrence of HCC.

Based on the above research findings, preS2-mutant LHBS may serve as a potential serum biomarker for anticipating the occurrence of HCC in CHB patients, as well as the heightened likelihood of HCC recurrence following extensive liver resection. Moreover, when assessing the risk of HCC recurrence, the preS2-mutant LHBS is combined with other high-risk markers such as AFP and des-gamma-carboxy prothrombin to improve the precision of prediction of prognosticating HCC recurrence risk post-hepatectomy [194, 195]. However, its feasibility requires further investigations to validate.

5.3 Research-Stage Drugs for Functional or Complete Cure.

The prevalence of HCC in our nation is substantial, with HBV infection identified as the primary risk factor contributing to over 80% of cases of HCC [196]. Consequently, for HBV-related HCC patients, along with anti-tumor treatment, anti-viral treatment is also crucial to reducing the mortality and recurrence rate of HCC. At present, antiviral agents utilized in clinical practice are predominantly categorized into two groups: nucleos(t)ide analogues and interferon drugs. The mechanisms of action and characteristics associated with these drugs are detailed in Table 1. The primary objective of antiviral therapy for HBV-related HCC is to suppress viral replication to the fullest extent possible, impede disease advancement, and alleviate liver impairment resulting from viral replication [12]. In addition, the implementation of effective antiviral therapy for patients after radical HCC resection can reduce the risk of postoperative recurrence and prolong the overall survival of the patients [197].

**Table 1:** Anti-HBV drugs in clinical use

Agents	Mechanism	Main advantages	Resistance	Safety	FDA approval status*	First-line recommendation	References
Nucleos(t)ide analogues (Oral administration)							
Lamivudine(LAM/3TC)	Competitively inhibit HBV DNA polymerase, suppressing HBV replication	Low cost; high antiviral efficacy; the main drug for early HBV antiviral treatment	High (Approximately 70% in 5 years)	Good, with few adverse reactions	Approved in 1998	No	[202, 203]
Adefovir (ADV)	Competitively inhibit HBV DNA polymerase, suppressing HBV replication	Effective for LAM-resistant patients	Moderate (Approximately 30% in 5 years)	High nephrotoxicity	Approved in 2002	No	[204, 205]
Telbivudine (LdT)	Competitively inhibit HBV DNA polymerase, suppressing HBV replication	High antiviral efficacy; minimal impact on renal function	High (Approximately 60% in 5 years), rtM204I mutation is the main resistance site	High muscular toxicity	Approved in 2006	No	[206–208]
Entecavir (ETV)	Competitively inhibit HBV DNA polymerase, suppressing HBV replication	Potently inhibit HBV replication	Very low (Approximately 1% in 5 years)	Good	Approved in 2005	Yes	[209, 210]
Tenofovir disoproxil fumarate (TDF)	Competitively inhibit HBV DNA polymerase, suppressing HBV replication	Rapidly and sustainably suppress HBV DNA	Very low (Less than 1% in 5 years)	Long-term use can lead to nephrotoxicity, reduced bone density, and other adverse reactions	Approved in 2008	Yes	[211–213]
Tenofovir alafenamide fumarate (TAF)	Competitively inhibit HBV DNA polymerase, suppressing HBV replication	Equivalent to TDF's HBV DNA suppression; lower impact on nephrotoxicity and bone density than TDF	Very low (Less than 1% in 5 years, lower than TDF)	Long-term use has demonstrated minimal impact on nephrotoxicity and bone density	Approved in 2016	Yes	[214, 215]
Tenofovir amibufenamide (TMF)	Competitively inhibit HBV DNA polymerase, suppressing HBV replication	Equivalent to TDF and TAF's HBV DNA suppression; lower impact on nephrotoxicity and bone density than TDF and TAF	Very low (Less than 1% in 5 years)	Long-term use has an extremely low impact on nephrotoxicity and bone density, lower than TAF and TDF	Not approved	No	[216, 217]
Interferons (Subcutaneous administration)							
IFNα	Enhance the host immune response and promote the proliferation and functional activation of NK cells; inhibit HBV replication by reducing HBV RNA levels or inducing cccDNA degradation	HBeAg seroconversion rate and HBsAg clearance rate are relatively high	Not applicable	Poor tolerability, multiple adverse reactions	Approved in 1991	No	[218–220]
Peg-IFNα	Enhance the host immune response and promote the proliferation and functional activation of NK cells; inhibit HBV replication by reducing HBV RNA levels or inducing cccDNA degradation	HBeAg seroconversion rate and HBsAg clearance rate are relatively high; longer serum half-life, fewer administration times	Not applicable	Adverse reactions are numerous, including influenza-like syndrome, bone marrow suppression, and autoimmune diseases	Approved in 2005	Yes	[221–223]

*The information on FDA approval status is from www.fda.gov, accessed March 1, 2025

cccDNA, covalently closed circular DNA; HBeAg, heaptitis B e antigen; HBV, hepatitis B virus; HBsAg, hepatitis B surface antigen

Investigational treatments currently in the experimental stages include entry inhibitors, RNA interference agents, capsid assembly modulators, and immunomodulatory approaches. Entry inhibitors, functioning as an entry-blocking antiviral agent, inhibit the entry of HBV into host cells through competitive and irreversible binding to NTCP with the large envelope protein of HBsAg [198, 199] (Table 2). The pharmaceutical agents presently undergoing clinical evaluation include Bulevirtide, Hepalatide, and Burfiralimab. Drugs that impede viral assembly can be classified into two categories: nucleic acid polymers (NAPs) and capsid assembly modulators (CAMs). NAPs, such as REP-2139, REP-2165, GST-HG131, and LP-128, hinder the assembly or secretion of SVPs and effectively decrease the level of HBsAg [200, 201]. CAMs, such as Morphothiadin, GST-HG141, Canocapavir, ABI-H3733, and ABI-4334, interfere with capsid assembly through allosteric binding to the core dimer and thereby misdirect capsid formation and disrupt encapsidation of pgRNA. Antisense oligonucleotides.

**Table 2:** New anti-hepatitis B virus drugs and properties

Type	Mechanism	Agent name	Drug class	Administration route
Entry inhibitor: Bulevirtide (BLV)	Bind to and inactivate the NTCP*, inhibiting entry of HBV	Myrcludex-B	Peptide	Subcutaneous
		Hepalatide	Peptide	Subcutaneous
		Burfiralimab (IgG4)	Monoclonal Antibody	IV*
Nucleic acid polymers (NAPs)	Inhibit assembly and secretion of hepatitis B virus subviral particles	REP-2139	Nucleic Acid Polymer	IV/Subcutaneous
		REP-2165	Nucleic Acid Polymer	IV/Subcutaneous
		GST-HG131	Small Molecule	Oral
		LP-128	Small Molecule	Oral
SiRNA* agents	Bind to the complementary target mRNA, leading to the cleavage of mRNA	JNJ-3989 (ARO-HBV)	siRNA	Subcutaneous
		AB-729	siRNA	Subcutaneous
		Vir-2218 (BRII-835)	siRNA	Subcutaneous
		RBD1016	siRNA	Subcutaneous
		STSG-0002	Unknown	Unknown
Antisense oligonucleotides (ASOs)	Bind to complementary HBV RNA transcripts, resulting in cleavage by ribonuclease H	Bepirovirsen (GSK836, GSK3228836)	ASO	Subcutaneous
		AHB-137	siRNA	Subcutaneous
Capsid assembly modulators (CAMs)	Interfere with capsid assembly through allosteric binding to the core dimer and thereby misdirect capsid formation and disrupt encapsidation of pgRNA	Morphothiadin (GLS4)	Small Molecule	Oral
		GST-HG141	Small Molecule	Oral
		Canocapavir (ZM-H1505R)	Small Molecule	Oral
		ABI-H3733	Small Molecule	Oral
		ABI-4334	Small Molecule	Oral
		JNJ-0440	Small Molecule	Oral
		ALG-000184	Small Molecule	Oral
Gene editing agents	Target by CRISPR-Cas9* to base edit cccDNA	EBT107	CRISPR	IV
	Target the HBV cccDNA with a sequence-specific ARCUS nuclease to eliminate HBV	PBGENE-HBV	CRISPR	IV
TLR7* agonists	Enhance the innate immune response by activating TLR7	RG7854 (RO7020531)	Small Molecule	Oral
		JNJ-4964 (AL-034/TQ-A3334)	Small Molecule	Oral
TLR8 agonist	Improve the innate immune response by activating TLR8	Selgantolimod (GS9688)	Small Molecule	Oral
		HRS9950	Small Molecule	Oral
		CB06-036 (CB06)	Unknown	Unknown
Immune checkpoint inhibitors	Enhance the recognition and killing capability of T lymphocytes by specific binding the PDL1 protein	ASC22 (Envafolimab)	Monoclonal Antibody	IV/Subcutaneous
		GS4224	Monoclonal Antibody	Oral
		AB-101	Monoclonal Antibody	Oral
	Enhance the recognition and killing capability of T lymphocytes by specific binding the PD-1 protein	Cemiplimab	Monoclonal Antibody	IV
Therapeutic vaccines	Activate effector T cells to enhance specific immune responses and exert antiviral effects	Nasvac	Protein	Intranasal/Subcutaneous
		HepTcell	Peptide	Subcutaneous
		vvx001	Peptide	Subcutaneous
		VBI-2601 (BRII-179)	Protein	Subcutaneous
		VTP-300	Viral Vector	Subcutaneous
		GSK3528869A	Protein	Subcutaneous
		CVI-HBV-002	Nucleic Acid	Intramuscular injection
		JNJ-64300535	Unknown	Subcutaneous

*The information on study status is from ClinicalTrials.gov, accessed January 23, 2025

NTCP: sodium taurocholate co-transporting polypeptide; siRNA: small interfering RNA; CRISPR-Cas9: clustered regularly interspaced short palindromic repeats and associated Cas9 homing endonucleases; TLR: toll-like receptor; IV: intravenous injection

(ASOs) and small interfering RNA (siRNA) are two pharmacological agents that attenuate the expression of HBsAg by specifically targeting HBV RNA. ASOs, including Bepirovirsen (GSK836, GSK3228836) and AHB-137, specifically recognize and bind to the messenger RNA utilized by the HBV for replication and transcription of viral antigens, leading to its cleavage by ribonuclease H and subsequent reduction of viral protein synthesis, particularly the prompt decrease in HBsAg, which is crucial to chronic hepatitis B patients in attaining a functional cure [224–227]. The action mechanism of siRNA closely resembles that of ASOs, as both molecules bind to messenger RNA to facilitate mRNA cleavage, thereby diminishing the synthesis of viral proteins. Additionally, siRNA exhibits a prolonged duration of action compared to GSK836. Promising results have been observed in clinical phase I and II trials, showing substantial efficacy and relatively slow post-treatment rebound, suggesting a potential approval for future therapeutic use [228–230]. It includes: JNJ-3989(ARO-HBV), AB-729, Vir-2218(BRII-835), RBD1016, and STSG-0002. These methodologies remain in the preclinical research phase. Notably, EBT107 is designed to target covalently closed circular DNA (cccDNA) through base editing using the CRISPR-Cas9 system, which consists of clusters of regularly interspaced short palindromic repeats and the associated Cas9 homing endonuclease. Additionally, PBGENE-HBV aims to target HBV cccDNA using a sequence-specific arc nuclease for the purpose of eradicating HBV.

Prior research showed that HBsAg could impair the immune response processes, leading to a compromised ability to exert normal anti-virus and anti-tumor immune functions. As a result, there is ongoing investigation into immunomodulatory treatments aimed at restoring and enhancing HBV-specific immune responses through the activation or replacement of endogenous immunity. These therapies include drugs designed to stimulate innate immune responses such as TLR 7 agonists, TLR8 agonists, and retinoic acid-inducible gene 1 agonists,

Which are constantly undergoing development and evaluation, the exploration of antibodies targeting the PD-1 axis or small molecule checkpoint inhibitors, and novel therapeutic vaccines [231–233]. TLR7 agonists, such as RG7854 (RO7020531) and JNJ-4964, augment the innate immune response of the host through the activation of TLR7. Similarly, TLR8 agonists, including Selgantolimod (GS9688), HRS9950, and CB06-036 (CB06), enhance innate immune responses by targeting TLR8. Additionally, immune checkpoint inhibitors, such as ASC22 (Envafolimab), GS4224, AB-101, and Cemiplimab, improve the recognition and cytotoxic capabilities of T lymphocytes by specifically binding to PD-L1 or PD-1 proteins. Therapeutic vaccines, such as Nasvac, HepTcell, vvx001, VBI-2601 (BRI-179), VTP-300, and GSK3528869A, augment specific immune responses and suppress antiviral effects through the activation of effector T cells. In addition to elucidating the primary mechanisms and characteristics of antiviral drugs, we have provided a comprehensive summary of clinical trials for investigational anti-HBV drugs, focusing on three aspects: trial phase, trial design, and primary outcome measures (Table 3). It is our aspiration that this work will contribute to the overarching objective of achieving HBsAg loss.

**Table 3:** Summary of clinical trials of anti-hepatitis B virus drugs under development

Drug	Phase	Trial design	Primary outcome measures
Myrcludex-B	Phase III (hepatitis D)	Monotherapy or combined with PEG-IFN alpha	HDV RNA decrease or detectable, ALT normalization
Hepalatide	Phase II	Monotherapy or combined with TAF and PEG-IFN	HDV RNA level, Proportion of subjects with negative conversion of HBsAg
Burfiralimab (IgG4)	Phase II	Monotherapy or combined with oral antiviral agent	Change in HBsAg from the baseline at 24 weeks (log10 IU/mL)
REP-2139	Phase II	Monotherapy and combined with NUCs or immunotherapy	Safety and tolerability, Efficacy (serum HBsAg, Number of patients with controlled HBV infection following treatment)
REP-2165	Phase II	Combination with NUCs	Number of patients with adverse events, reduction of serum HBsAg, and controlled HBV infection following treatment
GST-HG131	Phase II	Combined with NAs therapy	Change from Baseline in HBsAg levels, Safety assessment
LP-128	Phase I	Dose escalation	Area under the plasma concentration–time curve (AUC), Apparent terminal phase half-life (T1/2), Maximum observed plasma concentration (Cmax), and Adverse events of LP-128
JNJ-3989 (ARO-HBV)	Phase II	Combined with NUCs and PD-1 inhibitor	Percentage of Participants who Achieve HBsAg Seroclearance
AB-729	Phase II	Combined with NUCs and Durvalumab	Safety and tolerability, HBsAg levels, Soluble immune marker levels in plasma
Vir-2218 (BRII-835)	Phase II	Combined with BRII-179 and PEG-IFNα	Percentage of achieving HBsAg seroclearance
RBD1016	Phase II	Dose escalation	Safety (number and percentage of adverse events), Efficacy (the maximum decline of HBsAg level)
STSG-0002	Phase II	Unknown	Early-stage
Bepirovirsen (GSK836, GSK3228836)	Phase III	Combined with NA	Number of participants achieving functional cure with baseline HBsAg ≤ 3000 IU/mL
AHB-137	Phase I	Dose escalation	Safety, Tolerability, Pharmacokinetics, and Preliminary Efficacy
Morphothiadin (GLS4)	Phase II	Combined with RTV and ETV	The value of serum HBsAg decreased from baseline, Safety, Tolerability
GST-HG141	Phase II	Monotherapy	Adverse events and laboratory abnormalities, Pharmacodynamics and Pharmacokinetics
Canocapavir (ZM-H1505R)	Phase II	Dose escalation	Safety and tolerability, Pharmacokinetics, Pharmacodynamics
ABI-H3733	Phase I	Monotherapy	Safety, Pharmacokinetics, and Antiviral activity
ABI-4334	Phase I	Dose escalation	Safety, Pharmacokinetics, Antiviral activity
JNJ-0440	Phase I	Monotherapy	Safety, Tolerability, and Pharmacokinetics
ALG-000184	Phase I	Combined with Carbamazepine or Itraconazole	Safety, Tolerability, Pharmacokinetics and Drug-drug interaction
EBT107	Preclinical	Gene Editing	Early testing
PBGENE-HBV	Preclinical	Gene Editing	Early testing
RG7854 (RO7020531)	Phase II	Multiple Combination Therapies	Percentage of participants with Hepatitis B Surface Antigen loss
JNJ-4964 (AL-034/TQ-A3334)	Phase I	Monotherapy, Dose escalation	Safety, Tolerability and Pharmacokinetics
Selgantolimod (GS9688)	Phase II	Combination Therapies	Proportion of participants who achieve functional cure
HRS9950	Phase II	Dose escalation	Change in mean log10 serum hepatitis B surface antigen levels from baseline
CB06-036 (CB06)	Phase I	Dose escalation	Safety and Tolerability
ASC22 (Envafolimab)	Phase II	Dose escalation	Decreased HBsAg levels, the number of patients with ≥ 0.5log reduction in HBsAg log10IU/mL
GS4224	Phase I	Unknown	Early-stage
AB-101	Phase I	Dose escalation	Safety, Tolerability
Cemiplimab	Phase II	Unknown	Early-stage
Nasvac	Phase III	Combined with PEG-IFNα	Virological and or biochemical response
HepTcell	Phase II	Unknown	Early-stage
VBI-2601 (BRII-179)	Phase II	Combined with VIR-2218 and IFN-α	Sustained HBsAg loss, Safety
VTP-300	Phase II	Combined with Nivolumab	Safety, Tolerability and Immunogenicity
GSK3528869A	Phase II	Combined with NAs	Safety, Efficacy and Immune response
CVI-HBV-002	Phase I	Monotherapy	Safety, Reactogenicity and Immunogenicity
JNJ-64300535	Phase I	Combination Therapies	Reduction of at least 2 log10 international units per milliliter (IU/mL) in HBsAg levels from baseline

*The information on study status is from ClinicalTrials.gov, accessed January 23, 2025

PEG-IFN: polyethylene glycol interferon; ALT: alanine aminotransferase; NUCs or NAs: nucleos(t)ide analogs; HBsAg: Hepatitis B Surface Antigen; RTV: Ritonavir; ETV: Entecavir

## Conclusions and perspectives

HBsAg is significantly implicated in the occurrence, progression, and postoperative recurrence of HBV-associated HCC, influencing various cellular processes such as proliferation, ER stress, cell cycle regulation, metabolic reprogramming, and immune suppression. A thorough comprehension of the molecular mechanisms underlying HBsAg is essential for providing the theoretical basis for the diagnosis and therapeutic strategies of HBsAg-associated HCC. Studies have shown that the imbalance and misfolding of surface protein synthesis can lead to the retention in the ER of hepatocytes, subsequently causing ER stress, thereby inducing oxidative DNA damage and genomic instability, and also causing persistent sexual inflammation, aneuploidy production, and metabolic disorders [57, 58]. In addition, HBsAg can activate liver stem cell populations, which up-regulates the expression of CSC markers Epcam, CD 133, and CD117, ultimately promoting the malignant transformation of liver cells [126, 127]. HBsAg also induce the aberrant life activities of liver cells and the occurrence of HCC through activating transcription factors such as AP-1 and NF-κB, regulating the expression of proteins such as pyruvate kinase isoform M2, GRP78, CypA, and ECHS1, or abnormally activating the multiple cytoplasmic conduction pathways such as the PKCα/Raf1/Src/PI3K/Akt pathway and c-Raf-1/MEK/Erk2 signaling pathway [45, 48, 75, 83, 234, 235]. Equally important, the HBV envelope protein also affects the immune response process, inducing immune tolerance and immune escape by impairing the functions of dendritic cells, NK cells, and macrophages, causing HCC cells to be in a suppressive immune environment, thus conducive to the progress of HCC [95, 98, 99, 101–103, 106, 107, 109, 110]. However, numerous studies on HBsAg have not comprehensively revealed the mechanism leading to HCC or have solely demonstrated the carcinogenic effects of HBsAg without delving into the specific mechanisms involved. This lack of thorough investigation hinders a comprehensive understanding of HBV surface antigens and their association with HCC. Consequently, it is necessary to conduct comprehensive and precise experimental research into HBsAg.

In recent years, there have been a growing number of studies on the tumor immune microenvironment and tumor metabolism. However, limited attention has been given to the effects of HBsAg on these aspects in HBV-related HCC. As a critical protein involved in HBV infection and carcinogenesis, further investigation into additional mechanisms influencing the development and progression of HCC is warranted. Furthermore, ongoing advances in clinical diagnosis and treatment strategies are driven by extensive research into underlying mechanisms. Measurement of HBsAg levels holds significant clinical reference value in guiding preventive medication for HCC, determining optimal timing for drug administration, assessing therapeutic efficacy, and discontinuing medication. Currently, the primary focus of clinical drug development lies in antiviral and anti-tumor treatments, but antiviral drugs are not 100% effective. For HBsAg-positive patients who cannot prevent the expression of viral proteins, it may be beneficial to assess the potential efficacy of drugs that inhibit carcinogenic pathways activated by HBsAg. Developing treatment strategies can be considered based on the aberrantly expressed genes or proteins induced by HBsAg, such as targeted gene editing, specific protein receptor inhibitors, and targeting blockade of key transduction factors in oncogenic pathways. Additionally, combining the above approaches with conventional anti-tumor therapies may help decelerate tumor progression and extend patient survival.

## Data Availability

No datasets were generated or analysed during the current study.

## Abbreviations

AFP: Alpha-fetoprotein
AJCC: American joint committee on cancer
ALT: Alanine aminotransferase
AP-1: Activator protein 1
ASK1: Apoptotic signal-regulating kinase 1
ATF: Activator transcription factor
cccDNA: Covalently closed circular DNA
CHB: Chronic hepatitis B
CRISPR-Cas9: Clustered regularly interspaced short palindromic repeats and associated Cas9 homing endonucleases
CSC: Cancer stem cell
ECHS1: Enoyl-CoA hydratase short-chain 1
ER: Endoplasmic reticulum
Erk 2: Extracellular signal-regulated kinase 2
ETV: Entecavir
FOXP3: Forkhead box protein P3
GGH: Ground glass hepatocytes
GRP78: Glucose-regulated protein 78
HBV: Hepatitis B virus
HBsAg: Hepatitis B surface antigen
HCC: Hepatocellular carcinoma
HIF-1α: Hypoxia-inducible factor 1 alpha
hTRET: Human telomerase reverse transcriptase
IFN: Interferon
IRE1α: Inositol-requiring enzyme-1α
IV: Intravenous injection
JAB 1: Jun activation domain binding protein-1
JNK: C-Jun N-terminal kinase
LEF-1: Lymphoid enhancer-binding factor 1
LHB: Large envelope protein
MEK: Mitogen-activated extracellular signal-regulated kinase
MHB: Middle envelope protein
mTOR: Mammalian target of rapamycin
NAs: Nucleos(t)ide analogs
NF-κB: Nuclear factor kappa B
NK: Natural killer
NTCP: Sodium taurocholate co-transporting polypeptide
NUCs: Nucleos(t)ide analogs
ORF: Open reading frame
PD-1/PD-L1: Programmed cell death protein 1/programmed death-ligand 1
PEG-IFN: Polyethylene glycol interferon
PERK: Protein kinase R-like endoplasmic reticulum kinase
pgRNA: Pre-genomic RNA
PI3K: Phosphoinositide-3-kinase
PKC: Protein kinase C
PKM2: Pyruvate kinase isoform M2
RIDD: Regulated IRE1-dependent decay
RTV: Ritonavir
SHB: Small envelope protein
siRNA: Small interfering RNA
STAT 1: Signal transducers and activators of transcription
SVP: Subviral particles
TLR: Toll-like receptor
TNF-α: Tumor necrosis factor-α
TRAF2: Tumor necrosis factor receptor-associated factor 2
UPR: Unfolded protein response
VEGFA: Vascular endothelial growth factor-A
XBP1: X-box binding protein-1
